# Reliability and validity of sit-to-stand test protocols in patients with coronary artery disease

**DOI:** 10.3389/fcvm.2022.841453

**Published:** 2022-08-25

**Authors:** Zheng Wang, Jianhua Yan, Shu Meng, Jiajia Li, Yi Yu, Tingting Zhang, Raymond C. C. Tsang, Doa El-Ansary, Jia Han, Alice Y. M. Jones

**Affiliations:** ^1^Department of Sport Rehabilitation, School of Kinesiology, Shanghai University of Sport, Shanghai, China; ^2^Department of Cardiology, Xinhua Hospital Affiliated to Shanghai Jiao Tong University School of Medicine, Shanghai, China; ^3^Department of Physiotherapy, MacLehose Medical Rehabilitation Center, Hospital Authority, Hong Kong, China; ^4^Department Nursing and Allied Health, Swinburne University of Technology, Melbourne, VIC, Australia; ^5^Department of Surgery, Melbourne Medical School, University of Melbourne, Melbourne, VIC, Australia; ^6^Department of Physiotherapy, College of Rehabilitation Sciences, Shanghai University of Medicine and Health Sciences, Shanghai, China; ^7^Department of Physiotherapy, School of Health and Rehabilitation Sciences, The University of Queensland, Brisbane, QLD, Australia

**Keywords:** coronary artery disease, sit-to-stand, reliability, validity, discriminative ability, 6-minute walk test

## Abstract

**Background:**

Sit-To-Stand (STS) tests are reported as feasible alternatives for the assessment of functional fitness but the reliability of these tests in people with coronary artery disease (CAD) has not been reported. This study explored the test-retest reliability, convergent and known-groups validity of the five times, 30-sec and 1-min sit-to-stand test (FTSTS test, 30-s STS test and 1-min STS test respectively) in patients with CAD. The feasibility of applying these tests to distinguish the level of risk for cardiovascular events in CAD patients was also investigated.

**Methods:**

Patients with stable CAD performed a 6MWT and 3 STS tests in random order on the same day. Receiver operating characteristic (ROC) curve analyses were conducted using STS test data to differentiate patients with low or high risk of cardiovascular events based on the risk level determined by distance covered in the 6MWT as > or ≤ 419 m. Thirty patients repeated the 3 STS tests on the following day.

**Results:**

112 subjects with diagnoses of atherosclerosis or post-percutaneous coronary intervention, or post-acute myocardial infarction (post-AMI) participated in the validity analysis. All 3 STS tests demonstrated moderate and significant correlation with the 6MWT (coefficient values *r* for the FTSTS, 30-s STS and 1-min STS tests were−0.53, 0.57 and 0.55 respectively). Correlations between left ventricular ejection fraction (LVEF) and all STS tests and between 6MWT and LVEF were only weak (*r values* ranged from 0.27 to 0.31). Subgroup analysis showed participants in the post-AMI group performed worse in all tests compared to non-myocardial infarction (non-MI) group. The area under the curve (AUC) was 0.80 for FTSTS (sensitivity: 75.0%, specificity: 73.8%, optimal cut-off: >11.7 sec), and the AUC, sensitivity, specificity and optimal cut-off for 30-s STS and 1-min STS test were 0.83, 75.0%, 76.2%, ≤ 12 repetitions and 0.80, 71.4%, 73.8%, ≤ 23 repetitions respectively. The intraclass correlation coefficients (ICC) for repeated measurements of the FTSTS, 30-s STS and 1-min STS tests were 0.96, 0.95 and 0.96 respectively, with the minimal detectable change (MDC_95_) computed to be 1.1 sec 1.8 repetitions and 3.9 repetitions respectively.

**Conclusions:**

All STS tests demonstrated good test-retest reliability, convergent and known-groups validity. STS tests may discriminate low from high levels of risk for a cardiovascular event in patients with CAD.

## Introduction

Coronary artery disease (CAD) is the leading cause of death worldwide and accounts for a significant disease burden in developing countries (1, 2). Cardiac rehabilitation (CR) is supported by evidence as key to CAD management and functional capacity is often a clinical outcome used to reflect the success of a CR programme (3). The 6-min walk test (6MWT) has been used as a prognostic tool to predict the risk of myocardial infarct, heart failure and death in CAD patients (4). The 6MWT demands no expensive or complicated equipment, however it does require a corridor which is at least 30 meters in length, and the full assessment procedure takes at least 20 min. Further, patient performance can be confounded by the motivation and unintentional encouragement provided by the operators (5). In contrast, the sit-to-stand (STS) test requires a chair and minimal space. It was first introduced in 1985 for the evaluation of lower limb muscle strength (6). Recently, an incremental STS test protocol was reported to be highly correlated with peak oxygen consumption during an exercise test in healthy individuals (7). STS tests are now considered feasible alternative tests for the assessment of functional fitness in older adults and in various patient populations (8–10).

Tests or measures in clinical medicine are normally used for three purposes: discriminate between patient characteristics, predict an outcome or inform the prognosis of a condition, and evaluate change over time or change after certain intervention (11). The STS tests have been used to discriminate levels of functional capacity in people with chronic respiratory illness (12, 13) and have also been used to evaluate the effect of pulmonary rehabilitation in people with chronic obstructive pulmonary disease (COPD) (14). The 1-min STS test was shown to be a strong predictor of 2-year mortality in people with COPD (15).

While the reliability and discriminative properties of various STS test protocols, including the five times sit-to-stand (FTSTS) test, 30-sec STS (30-s STS) test and 1-min STS (1-min STS) test, have been reported in different populations (16–18), a gold standard for testing the criterion validity of STS tests in discriminating levels of functional capacity in people with CAD is not yet available. There is therefore a need to establish the construct (convergent and known-groups) validity of STS tests in this population. Further, while STS test performance in patients with heart failure (19) and in patients enrolled in CR (20) have been reported, comparison of the reliability of various STS test protocols in patients with CAD has not been reported.

The focus of this current study is to examine the discriminative properties of the FTSTS, 30-s STS and 1-min STS tests. The primary objective of this current study is to examine the test-retest reliability, convergent and known-groups validity of the 3 STS tests. Convergent validity was examined through investigation of the correlation between STS test scores and the distance covered by the 6MWT, a valid test for measurement of functional capacity. Known-groups validity was examined through the evaluation of statistical and clinically important differences in STS tests of known groups, that is, participants with post-AMI and those without myocardial infarction (non-MI).

Following the assumption that STS tests are feasible alternative measurement tool for functional capacity, the secondary objective of the study was to explore the feasibility of using STS test protocols to discriminate the level of risk of cardiovascular events in people with CAD. Categorization of risk of cardiovascular events was adopted from a previous report, that patients who covered ≤ 419 m during a 6MWT were classified at high risk of cardiovascular events (4).

## Materials and methods

### Study design and participants

A cross-sectional study (Chinese Clinical Trial Registry Number: 2000037435) was conducted from July 2020 to February 2021. Approval to conduct the study was granted by the Ethics Committee of Xinhua Hospital Affiliated to Shanghai Jiaotong University School of Medicine (Approval Number: XHEC-C-2020-078-1). Participants were considered eligible for enrolment if they were older than 18 years with CAD with a diagnosis of atherosclerosis (as confirmed by angiography), post percutaneous coronary intervention, or at least 6 months post-acute myocardial infarction (post-AMI). The exclusion criteria included: a) raised cardiac troponin I level, b) acute infectious disease, respiratory disease, uncontrolled metabolic disease or hypertension; c) with neuromusculoskeletal, cognitive disorders, visual or auditory dysfunction that would affect performance of STS tests; d) heart failure at Class III or IV of the New York Heart Association (NYHA) classification; e) having previously participated in a CR program.

### Procedures

Patients attending follow up at the Xinhua Hospital were screened for eligibility for this study. Eligible patients were invited to participate, with the nature of the study explained and written informed consent obtained from each patient prior to commencement of the study. The socio-demographic characteristics of each participant were then recorded.

Participants were asked to complete a 6MWT, FTSTS, 30-s STS and 1-min STS tests at random order on the same day. All tests were performed once only without any trial runs. This was deemed appropriate as test-retest reliability of FTSTS test was shown to be reliable with a single evaluation session (17). The order of testing was written in a card placed in a sealed envelope and the participants were asked to draw an envelope with the concealed order enclosed. A rest period of 30 min was set between the 6MWT and any of the STS tests, and all participants were given a 5-min rest period between each STS test protocol (21). Blood pressure (BP), heart rate (HR) and level of fatigue (using a CR-10 Borg scale) were recorded before and immediately after each test. Any adverse events were documented. All tests were conducted by the same physiotherapist.

All participants were also invited to draw from another sealed envelope which determined whether they were invited to return on the follow day to repeat the 3 STS tests.

#### Sit-to-stand test protocols

A chair without arms, with rubber tips on the legs, a hard seat of fixed height 46 cm was positioned against a wall. All 3 STS test protocols required the participant to commence in a seated position with the feet resting flat and ankle joints in the neutral position. All participants were given the instruction to perform the sit-to-stand task as quickly as possible with their arms crossed over the chest and hands on the shoulders (8). A standard STS movement was viewed as the legs being fully straightened when the stand phase concluded and the hip landing firmly on the chair when seated. A test would be terminated if the participant required assistance or was unable to complete the movement. No encouragement was provided during any STS test protocol.

The time for the participant to complete the STS movement five times was recorded for the FTSTS test (12). For 30-s and 1-min STS tests, subjects were required to perform as many STS movements as possible within 30 sec (22) or 1 min, respectively. The number of STS movements completed within the time required was recorded, as appropriate.

#### 6MWT

The 6MWT was conducted following the American Thoracic Society guidelines for the 6MWT (23). All participants completed the 6MWT on a 30-meter flat corridor.

### Statistical analysis

Data analyses were performed using the IBM SPSS Statistics 25.0 for Windows (IBM Corp., Armonk, NY) and the MedCalc 18.2.1 (Ostend, Belgium). Continuous variables were reported as mean and standard deviation (SD), and categorical data were shown as a number and percentage. The Kolmogorov-Smirnov test was used to check the normality of data distribution. Test-retest reliability, defined as consistency of the STS test scores recorded in the two separate days, was determined by the intraclass correlation coefficient (ICC) calculated by a two-way mixed model with absolute agreement method (24). The ICC was considered as poor, fair, good and excellent with values of <0.5, 0.5 to 0.75, 0.75 to 0.9, >0.9 respectively (25). Bland-Altman plots were constructed to display the distribution difference of repeated measurements and calculate the limits of agreement (LoA) *via* mean difference ± 1.96 SD of difference (26). Standard error of measurement (SEM) and a minimal detectable change with 95% confidence intervals (MDC_95_) were calculated with the formulae SD$\sqrt{1-ICC}$ and SEM × $\sqrt{}2\times$1.96 respectively, to estimate the measurement variability (27). Pearson's or Spearman's correlation coefficients were used to measure the magnitude of correlation between STS tests, 6MWT and LVEF, depending on the data distribution. The correlation was considered “negligible to weak,” “moderate,” and “well-accepted” with values <0.3, 0.3 to 0.6, and >0.6 respectively (28, 29). One way to support the convergent validity of the STS tests was a satisfactory correlation value between the STS scores and outcomes recorded with another tool valid for measurement of functional capacity, thus it was hypothesized that the correlation value (r) generated between STS tests and recorded 6MWD should be at least 0.5. Independent Samples *t* test or Mann-Whitney test was used to explore the known-groups validity through analysis of the differences in all tests between groups of patients with non-MI and post-AMI. Based on previous reported values of minimal clinically important differences (MCID) in STS tests in subjects with COPD, the between-group mean difference (MD) in STS tests was hypothesized to be ≥1.3s for the FTSTS test (12), ≥2 repetitions for 30-s STS test (14) and ≥3 repetitions for the 1-min STS test (30). Chi-square test was used to compare distributions of gender, coronary artery lesions, cardiovascular risk factors and type of medications between subgroups.

Lastly, if STS tests are deemed feasible alternatives to 6MWT in assessment of functional capacity in people with CAD, we wish to explore whether STS tests were able to discriminate participants with high and low risk of cardiovascular events, thus, receiver operating characteristic (ROC) curve analyses (31) were performed. The area under the curve (AUC) of the ROC curve would indicate the discriminative ability of the STS tests to differentiate the risk of cardiovascular events. The selection of the optimal cut-off scores for the STS tests was based on principle of minimizing the misclassification with minimum absolute difference between sensitivity and specificity (32). Based on a previous report by Beatty and colleagues, the “cut-off” value adopted in this study to differentiate participants with low or high risk of cardiovascular events was 419 m from the 6MWT (4). The AUC of all STS tests were compared. All statistical tests were two-tailed with the level of significance set at 0.05.

### Sample size estimation

With two observations per subject, to achieve 80% power to detect an estimated ICC of 0.8 under the alternative hypothesis, and with the ICC estimated as 0.5 under the null hypothesis, with a significance level of 0.05, the minimum sample size for the STS test-retest reliability was computed as 22. For the known-groups validity testing, the sample size estimation was based on a hypothesized effect size index of 0.6 in testing the mean STS test difference between groups of non-MI and post-AMI patients, using a two-tailed independent *t*-test with a level of significance of 0.05 and statistical power of 0.8. A minimum of 90 patients was required. In the ROC curve analyses of STS tests for differentiating patients with low or high risk of cardiovascular events, a minimum sample of 82 patients was required to achieve 80% power to detect a difference of 0.15 between the AUC of the ROC curve analysis under the null hypothesis of 0.70 and an AUC under the alternative hypothesis of 0.85 using a two-sided z-test at a significance level of 0.05. All the sample size estimations were performed using the PASS 15.0.5 (NCSS, Kaysville, Utah, USA).

## Results

### Subjects characteristics

A total of 112 patients (mean age 63.9 ± 8.8 years, male 69%) were included in the study and the mean age of the 30 patients participated in the reliability tests was 64.3 ± 7.3 years, (males 53%). The number of post-AMI and non-MI participants was 48 and 64 respectively. Demographic characteristics of all patients are summarized in Table 1. Table 2 displays the HR, BP and level of fatigue at pre and immediately post STS tests and 6MWT. There was no adverse event during all tests. In the post-AMI group, 1 patient required a permanent pacemaker, 2 patients received coronary arterial bypass grafting operations.

**Table 1:** Demographics characteristics of the participants.

**Characteristic**	**Validity study** **(*n* = 112)**	**Reliability study (*n* = 30)**
Age (years)	63.9 ± 8.8	64.3 ± 7.3
**Gender**		
Male	77 (69%)	16 (53%)
Female	35 (31%)	14 (47%)
BMI (kg/m^2^)	24.6 ± 3.5	24.8 ± 3.5
**Diagnosis**		
Atherosclerosis	34	10
post-PCI post AMI	30 48	10 10
**Coronary artery lesions**		
Single-vessel	41 (37%)	14 (47%)
Double-vessel	24 (21%)	7 (23%)
Triple-vessel	47 (42%)	9 (30%)
**Cardiovascular risk factors**		
Hypertension	69 (62%)	17 (57%)
Diabetes	43 (38%)	13 (43%)
Hyperlipidaemia	64 (57%)	15 (50%)
Smoking	71 (63%)	20 (67%)
**Blood tests**		
FBG (mg/dl)	107.91 ± 26.20	110.13 ± 33.43
TC (mg/dl)	144.46 ± 39.04	140.99 ± 25.84
TG (mg/dl)	164.64 ± 144.30	190.14 ± 139.95
HDL (mg/dl)	39.78 ± 10.89	37.51 ± 10.50
LDL (mg/dl)	79.39 ± 31.51	77.03 ± 21.50
NT-proBNP (pg/ml)	283.90 ± 597.48	322.90 ± 811.73
**Current medications**		
Antiplatelet agents	102 (91%)	27 (90%)
ACEI/ARB	48 (43%)	11 (37%)
β-Blocker	108 (96%)	28 (93%)
Lipid-lowing agents	107 (96%)	29 (97%)
Hypoglycemic agents	36 (32%)	10 (33%)

PCI, percutaneous coronary intervention; post-AMI, post-acute myocardial infarction; BMI; body mass index; FBG, fasting blood glucose; TC, total cholesterol; TG, triglycerides; HDL, high density lipoprotein; LDL, low density lipoprotein; NT-proBNP, N-terminal pro b-type Natriuretic Peptide; ACEI, angiotensin-converting enzyme inhibition; ARB, angiotensin receptor blocker.

**Table 2:** Hemodynamic data and fatigue score at pre- and immediately post-tests (*n* = 112).

		**6MWT**	**FTSTS**	**30-s STS**	**1-min STS**	***p*-value^Δ^ across groups**
HR (beats/min)	pre post	72.9 ± 8.3 85.9 ± 8.4	71.1 ± 6.8 73.8 ± 7.8	72.7 ± 8.6 79.6 ± 9.2	72.3 ± 8.2 87.5 ± 8.9	*p* = 0.35
	% change	+17.8%	+3.8%	+9.5%	+21.0%	*p* <0.001
*p*-value^*^		*p* <0.001	*p* <0.001	*p* <0.001	*p* <0.001	
SBP (mmHg)	pre post	125.0 ± 12.5 146.8 ± 14.1	124.9 ± 13.3 130.4 ± 12.4	125.3 ± 13.5 139.4 ± 16.3	128.2 ± 12.6 149.3 ± 14.8	*p* = 0.18
	% change	+17.4%	+4.4%	+11.3%	+16.5%	*p* <0.001
*p*-value^*^		*p* <0.001	*p* <0.001	*p* <0.001	*p* <0.001	
DBP (mmHg)	pre post	73.0 ± 9.7 81.5 ± 8.6	73.3 ± 8.8 73.4 ± 9.3	74.3 ± 8.4 83.0 ± 8.7	75.3 ± 7.5 84.5 ± 8.8	*p* = 0.20
	% change	+11.6%	0.1%	+11.7%	+12.2%	*p* <0.001
*p*-value^*^		*p* <0.001	*p* = 0.90	*p* <0.001	*p* <0.001	
Fatigue score	pre	0.2 ± 0.4	0.2 ± 0.5	0.2 ± 0.6	0.2 ± 0.5	*p* = 0.93
	post	4.0 ± 0.9	0.3 ± 0.6	2.4 ± 1.1	4.2 ± 1.7	*p* <0.001
*p*-value^*^		*p* <0.001	*p* = 0.05	*p* <0.001	*p* <0.001	

HR, heart rate, SBP, systolic blood pressure, DBP, diastolic blood pressure, 6MWT, six-minute walk test, FTSTS, five times sit-to-stand, 30-s STS, 30-second sit-to-stand, 1-min STS, 1-minute sit-to-stand. Mean diff, mean difference between pre and post values.

* p-value determined by paired t-test or Wilcoxon signed rank test; ^Δ^p-value determined by repeated measures ANOVA or Kruskal-Wallis H test.

### Relative and absolute reliability

The relative reliability for FTSTS, 30-s STS, 1-min STS tests were excellent with ICC values all above 0.95 between the two testing days in all STS tests. The minimum detectable change values with 95% confidence interval (MDC_95_) were 1.11 s, 1.79 repetitions and 3.86 repetitions, respectively (Table 3).

**Table 3:** Relative and absolute reliability results of the three STS tests (*n* = 30).

	**ICC (95% CI)**	**1^st^ measure**	**2^nd^ measure**	**SEM**	**MDC_95_**	**95% LoA**
FTSTS (sec)	0.96 (0.92–0.98)	12.01 ± 2.04	11.85 ± 2.14	0.40	1.11	−0.96–1.26
30–s STS (repetitions)	0.95 (0.91–0.98)	13.07 ± 3.06	12.97 ± 3.02	0.65	1.79	−1.71–1.91
1–min STS (repetitions)	0.96 (0.91–0.98)	24.67 ± 6.69	25.10 ± 6.69	1.39	3.86	−4.31–3.44

ICC, intraclass correlation coefficient; 95% CI, 95% confidence interval; SEM, standard error of measurement; MDC_95_, minimum detectable change with 95% CI; LoA, 95% limits of agreement; FTSTS, five times sit–to–stand; 30–s STS, 30–sec sit–to–stand; 1–min STS, 1–min sit–to–stand.

### Convergent validity

**Figure 1:**
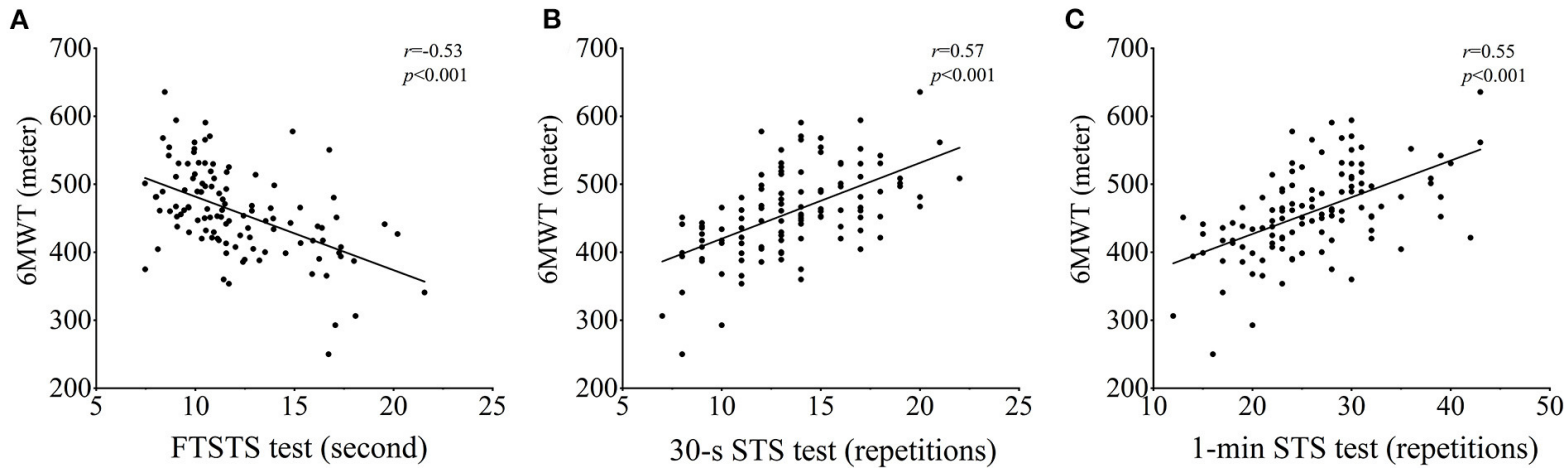
Correlations between 6MWT and the 3 STS tests. FTSTS test: five times sit-to-stand test, 30-s STS test: 30-sec sit-to-stand test, 1-min STS test: 1-min sit-to-stand test, 6MWT: six-min walk test.

Correlation between performance of 6MWT and the 3 STS tests were moderate (FTSTS test, *r* = −0.53, *p* < 0.001; 30-s STS test, *r* = 0.57, *p* < 0.001; 1-min STS test, *r* = 0.55, *p* < 0.001). Correlations between recorded LVEF and STS performance or with 6MWT were both weak (Table 4, Figure 1).

**Table 4:** Correlation analysis between STS tests with 6MWT and LVEF.

		**FTSTS**	**30–s STS**	**1–min STS**	**6MWT**
6MWT	*r*	*r_*s*_* = −0.53	*r_*s*_* = 0.57	*r_*p*_* = 0.55	–
	*p*	<0.001	<0.001	<0.001	–
LVEF	*r*	*r_*s*_* = −0.27	*r_*s*_* = 0.31	*r_*s*_* = 0.27	*r_*s*_* = 0.29
	*p*	0.003	0.001	0.004	0.002

FTSTS, five times sit–to–stand; 30–s STS, 30–sec sit–to–stand; 1–min STS, 1–min sit–to–stand; 6MWT, six–min walk test; LVEF, left ventricular ejection fraction.

r = Rho; r_p_ = Pearson correlation coefficient; r_s_ = Spearman correlation coefficient; p = p–value.

### Known-groups validity

**Figure 2:**
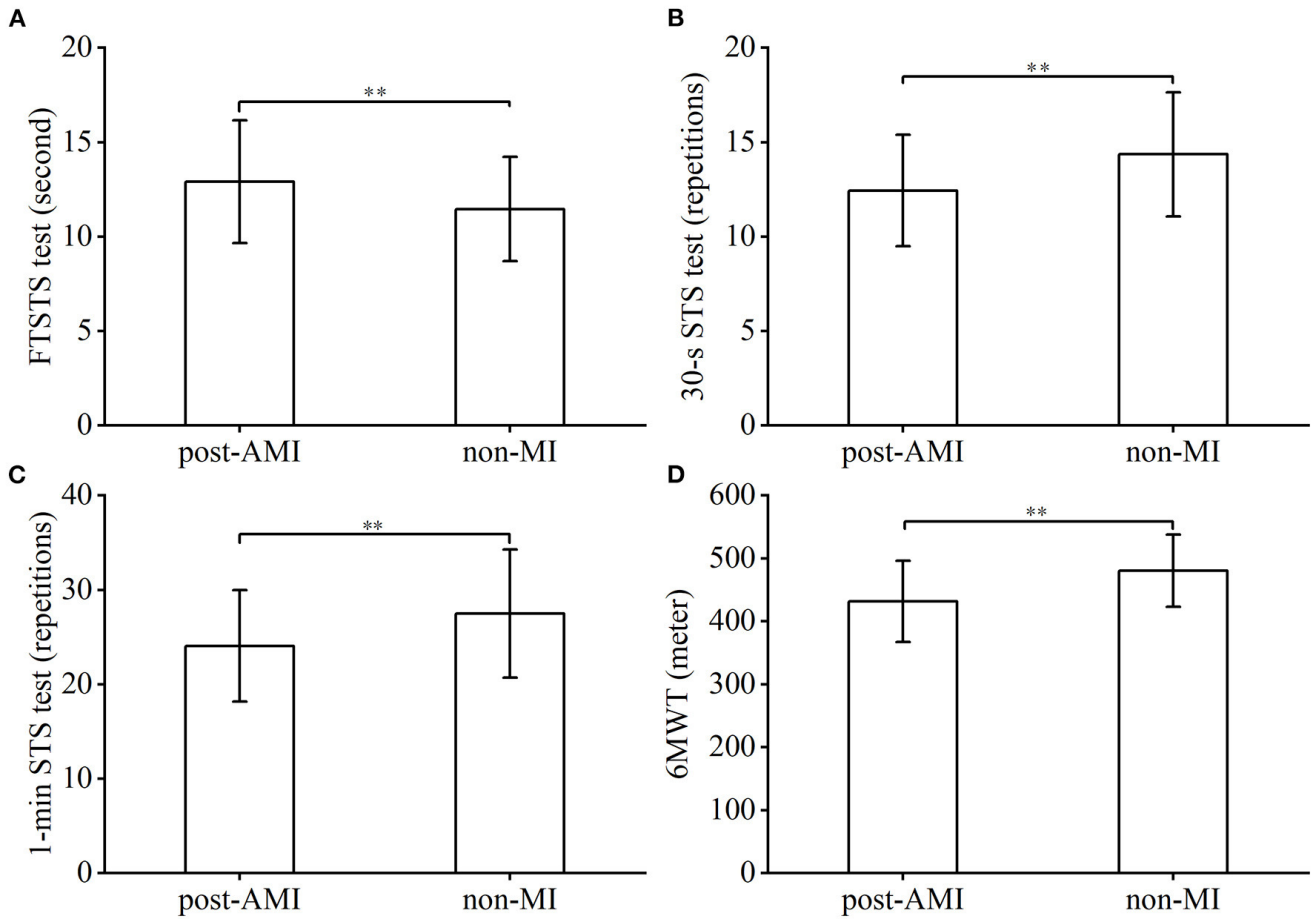
Intergroup comparison of STS tests and 6MWT performance. FTSTS test: five times sit-to-stand test, 30-s STS test: 30-sec sit-to-stand test, 1-min STS test: 1-min sit-to-stand test, 6MWT: six-min walk test, post-AMI: post-acute myocardial infarction. ** *p* < 0.01.

Table 5 displays results of known-groups validity analyses between patients in the post-AMI and non-MI groups. Patients with post-AMI required significantly longer time in completion of the FTSTS test, means difference (MD) and 95%CI being 1.4 s, 0.3 to 2.6, (*p* = 0.009), less repetitions in both the 30-s STS, MD−2.0, 95% CI: −3.1 to −0.7, (*p* = 0.002) and 1-min STS test MD−3.4, 95%CI: −5.9 to −1.0 (*p* = 0.006); the distance covered in the 6MWT (431.5 ± 64.6 vs. 480.3 ± 57.3, MD −48.8, 95% CI: −71.7 to −25.9, *p* < 0.001) was also less in this group (Table 5, Figure 2). These mean differences were similar or larger than the pre-specified differences with reference to the MCID values of the STS tests of patients with COPD.

**Table 5:** Comparison of STS tests and 6MWT performance between post–AMI and non–MI groups.

	**post–AMI**	**non–MI**	**Mean Difference**	**95% CI**	***p*–value**
FTSTS test (sec)	12.9 ± 3.3	11.5 ± 2.8	1.4	0.3 ~ 2.6	*P* = 0.009^**^
30–s STS test (repetitions)	12.4 ± 2.9	14.4 ± 3.3	−2.0	−3.1 ~−0.7	*p* = 0.002^**^
1–min STS test (repetitions)	24.1 ± 5.9	27.5 ± 6.8	−3.4	– 5.9 ~−1.0	*p* = 0.006^**^
6MWT (meter)	431.5 ± 64.6	480.3 ± 57.3	−48.8	−71.7 ~−25.9	*p* < 0.001^**^

post–AMI, post–acute myocardial infarction; 95% CI, 95% confidence interval; FTSTS test, five times sit–to–stand test; 30–s STS test, 30–sec sit–to–stand test; 1–min STS test, 1–min sit–to–stand test; 6MWT, six–min walk test.

** p < 0.01.

### ROC curve analysis

A total of 84 patients achieved more than 419 m in their 6MWT and were thus classified as low- risk cardiovascular event group (4), 28 patients who covered ≤ 419 m were in the high-risk group. ROC curve analysis illustrated that the AUC of FTSTS, 30-s STS,and 1-min STS tests were 0.80 (sensitivity: 75.0%, specificity: 73.8%, optimal cut-off: 11.7 s), 0.83 (sensitivity: 75.0%, specificity: 76.2%, optimal cut-off: 12 repetitions), and 0.80 (sensitivity: 71.4%, specificity: 73.8%, optimal cut-off: 23 repetitions) respectively (Table 6). There were no statistically significant differences in the AUC of the ROC curve analyses among the three STS tests.

**Table 6:** ROC analysis of discriminatory capability of STS tests on 6MWD over 419 m.

**Tests**	**AUC (95% CI)**	**optimal cut-off**	**Sensitivity**	**Specificity**	***p*-values**
FTSTS (second)	0.80 (0.71–0.87)	>11.7	75.0%	73.8%	*p* <0.001^**^
30-s STS (repetitions)	0.83 (0.75–0.89)	≤ 12	75.0%	76.2%	*p* <0.001^**^
1-min STS (repetitions)	0.80 (0.71–0.87)	≤ 23	71.4%	73.8%	*p* <0.001^**^

ROC, receiver operating characteristic; 6MWD, distance of six-min walk; AUC, area under the curve; FTSTS, five times sit-to-stand; 30-s STS, 30-sec sit-to-stand; 1-min STS, 1-min sit-to-stand.

** p < 0.01.

## Discussion

The use of FTSTS test has been shown to be an instrument of high reliability in healthy adults (33) as well as in people with spinal cord injury (34), hip arthroplasty (17) and in people with chronic obstructive pulmonary disease (12). The 1-min STS test and 30-s STS test were also shown to be reliable measures in people with pulmonary hypertension (22), total knee arthroplasty (35), with COPD (13) and cystic fibrosis (36). This current study is the first that compared all three STS tests in the same population of people with CAD. Findings of our study demonstrated excellent test-retest reliability for all 3 STS tests in this subject-cohort. Further, we found that compared to FTSTS and 30-s STS tests, the 1-min STS test induced the greatest changes in heart rate and SBP, and associated with the highest post-test fatigue score (Table 2). Amongst the 3 STS tests, changes induced by the 1-min STS test were closest to the physiological changes induced by the 6MWT. This suggests that the 1-min STS test is superior than 30-s STS and FTSTS tests if considered as an assessment tool for functional capacity in people with CAD. Our findings were in accord with a previous report which recommended the 1-min STS test to be a superior STS protocol for evaluation of functional capacity in subjects with COPD (8). Further, oxygen consumption was shown to be highest during a 1-min STS test compared to FTSTS in a cohort of patients with COPD (30). Although we have not measured oxygen consumption during the STS tests in our participants with CAD, we postulate that the pattern of oxygen uptake during STS tests would be similar in our subject cohort compared to those with COPD; further analysis is necessary to confirm this assumption.

As anticipated, findings of the current study illustrated moderate correlation between the 3 STS tests and the 6MWT, the correlation coefficient data of our study are similar to those previously reported in community-dwelling older adults (10) and in a cohort of patients with COPD (37). The correlation between LVEF and 6MWT and between LVEF and any STS tests was however only weak. This is not surprising. A lack of correlation between distanced covered in a 6MWT and ejection fraction was also reported in patients with heart failure (38). Exercise capacity was shown to be inversely associated with diastolic dysfunction but not associated with variation of left ventricular systolic function in people with LVEF within normal range (39). The LVEF of our patient cohort was all above 56%, it is therefore not surprising that there existed only a weak correlation between LVEF and exercise capacity (reflected by 6MWT or STS tests). While cardiac dysfunction partly contributes to poor exercise capacity, skeletal muscle fatigability plays an important mechanism in exercise tolerance (40); further, in patients with a normal ejection fraction, exercise capacity is associated with left ventricular dimensions (41), however, assessment of skeletal muscle fatigability and appraisal of left ventricular dimension was outside of the scope of this study.

The 6MWT has been suggested to be a prognostic tool for cardiovascular events in patients with stable CAD (4). Beatty and colleagues reported that patients in the lowest quartile of 6MWD of 87-419 m had 4 times the risk of cardiovascular events compared to those in the highest 6MWD quartile of 544-837 m. Based on Beatty and colleagues' work, we used 6MWD of 419 m to demarcate high or low-risk cardiovascular event groups. With ROC curve analysis of our data, we showed that the 3 STS tests demonstrated good discriminative ability to differentiate patients between high and low risk cardiovascular events. Results of this analysis suggest that for patients with CAD, those who required more than 11.7 sec to complete a FTSTS test, or those who completed <12 repetitions of STS sequence in 30 sec or <23 STS-repetitions in 1 min were subjected to higher risk of cardiovascular events.

The MDC_95_ data generated from this study illustrated the measurement errors on repeated measurements of the STS tests. The MDC_95_ value for FTSTS test was previously reported in patients with COPD to be 3.1s (12). Our study showed that the MDC_95_ value for FTSTS test in people with CAD was 1.1s. This suggests that the measurement error of FTSTS test was less in our cohort of participants with CAD compared to people with COPD. Similarly, MDC_95_ value for the 30-s STS test reported in subjects with COPD (42) was also higher than that generated in this study. The MDC_95_ of 1-min STS test reported by Crook and colleagues (30) was however smaller than our results (2.2 repetitions compared to 3.86 repetitions in our CAD cohort). Direct comparison between our study and previous reports however may not be appropriate. As the MDC_95_ in other reports were generated from tests conducted in patients with COPD, whose exercise capacity was limited by impairment of the ventilatory system, and our data were obtained from patients with CAD, whose functional capacity is often restricted by cardiac performance.

Smoking, hyperlipaemia, hyperglycaemia, and hypertension are known risks factors of cardiovascular disease (43). Table 1 shows that the rate of smoking is high amongst our participants. Unfortunately, China is the largest consumer of tobacco in the world (WHO-Tobacco in China) (44) and smoking prevalence is about 26.6% (45); there are more than 300 million smokers in China, this suggests that greater effort is required to foster public health strategies of smoking cessation.

In summary, this study showed that all 3 STS tests have excellent test-retest reliability. The MDC_95_ values reported in this study provide a useful guide for the versatile use of STS protocols. The relationship of the STS tests with 6MWT demonstrated that these tests have good convergent validity in assessment of functional capacity in patients with CAD. Known-groups validity of these STS test was illustrated through distinct performance level by non-MI and post-AMI groups. STS tests are easy to perform and can be used as an assessment as well as an exercise training tool. Lastly this study inferred that STS test can also be considered as a discriminative tool to predict high or low risk of cardiovascular events in patients with CAD.

### Limitations of the study

This study demonstrated a satisfactory correlation between 6MWD and the STS tests and proposed that STS tests can be considered as a useful tool to assess function capacity. While we have demonstrated similar haemodynamic responses between 6MWT and the STS test, we did not have appropriate equipment to measure oxygen consumption during the STS tests. Information on oxygen uptake during the STS test would provide a more detailed picture of physiological demands of STS tests.

High and low risk cardiovascular events was categorized using 6MWD of 419 m as the demarcation level. While the predictive validity of this assumption was proven (4), inclusion of cumulative biometric risk factors may provide a stronger profile of risks of cardiovascular events. Validity of an index which combines the 6MWD and biometric risk factors requires further investigation.

The focus of this study was to establish discriminative properties of the three STS tests; establishment of the evaluative and prognostic properties of these tests in people with CAD was outside the scope of this study. Further research in examining the evaluative and prognostic value of STS tests is warranted.

## Conclusion

This is the first study that compared FTSTS test, 30-s STS test, and 1-min STS test in patients with CAD. This study showed that all 3 STS tests had good test-retest reliability, convergent and known-groups validity in assessing functional capacity in CAD patients. All 3 STS tests demonstrated good discriminative property in distinguishing patients with high and low risk of cardiovascular events and as such may inform the individualized clinical management, targeted exercise programmes and rehabilitation in this patient population.

## Data availability statement

The original contributions presented in the study are included in the article/supplementary material, further inquiries can be directed to the corresponding authors.

## Ethics statement

The studies involving human participants were reviewed and approved by the Ethics Committee of Xinhua Hospital Affiliated to Shanghai Jiaotong University School of Medicine (Approval Number: XHEC-C-2020-078-1). The patients/participants provided their written informed consent to participate in this study.

## Author contributions

Study conceptualization: ZW, JY, SM, JL, JH, and AJ. Data curation: ZW, JY, SM, JL, YY, and TZ. Statistical analysis: RT and DE-A. Manuscript preparation and writing the first draft: ZW, JY, and SM. Writing the section of the manuscript: JL, YY, TZ, DE-A, RT, JH, and AJ. All authors contributed to manuscript revision and have read and approved the final version of the manuscript.

## Funding

This work was supported by the National Natural Science Foundation of China (No. 31870936).

## Conflict of interest

The authors declare that the research was conducted in the absence of any commercial or financial relationships that could be construed as a potential conflict of interest.

## Publisher's note

All claims expressed in this article are solely those of the authors and do not necessarily represent those of their affiliated organizations, or those of the publisher, the editors and the reviewers. Any product that may be evaluated in this article, or claim that may be made by its manufacturer, is not guaranteed or endorsed by the publisher.

## Abbreviations

CAD: coronary artery disease
CR: cardiac rehabilitation
6MWT: 6-min walk test
STS: sit-to-stand
FTSTS test: five times sit-to-stand test
30-s STS test: 30-sec sit-to-stand test
1-min STS test: 1-min sit-to-stand test
LVEF: left ventricular ejection fraction
post-PCI: post percutaneous coronary intervention
non-MI: non-myocardial infarction
post-AMI: post-acute myocardial infarction
BP: blood pressure
HR: heart rate
SD: standard deviation
ICC: intraclass correlation coefficient
LoA: limits of agreement
SEM: standard error of measurement
MDC_95_: minimal detectable change with 95% confidence interval
ANOVA: one-way analysis of variance
ROC: receiver operating characteristic
AUC: area under the curve
COPD: chronic obstructive pulmonary disease
6MWD: the distance of 6MWT
MCID: minimal clinically important difference
MD: mean difference.
